# Supplementary figures and images for: Temporal dynamics of neuroplasticity and neurodegeneration in the central auditory system following noise-induced hearing loss: a multimodal imaging and histological study
Source: Acta Neuropathol Commun. 2026 Jun 2;14:119. doi: 10.1186/s40478-026-02252-8 (PMC13227874; doi:10.1186/s40478-026-02252-8)

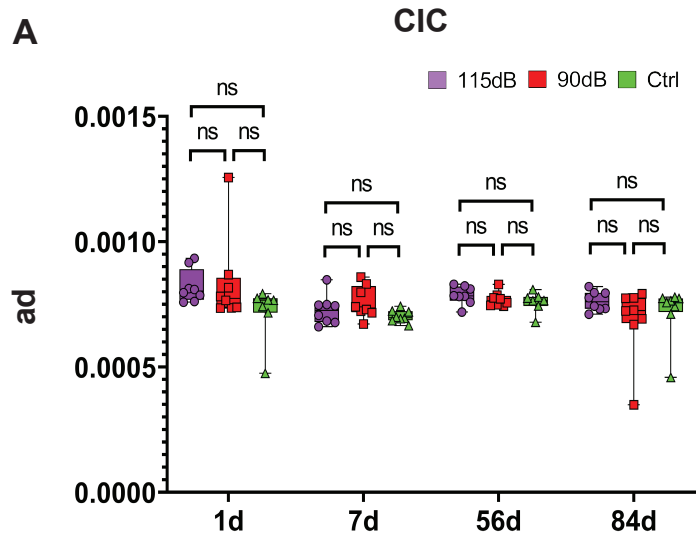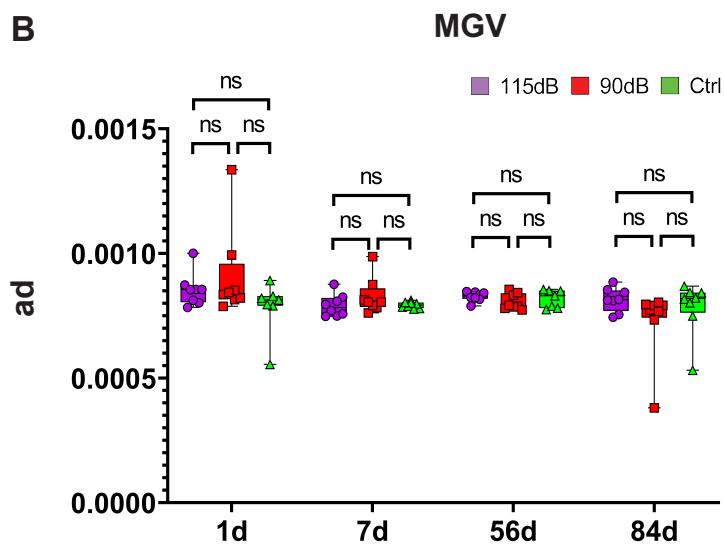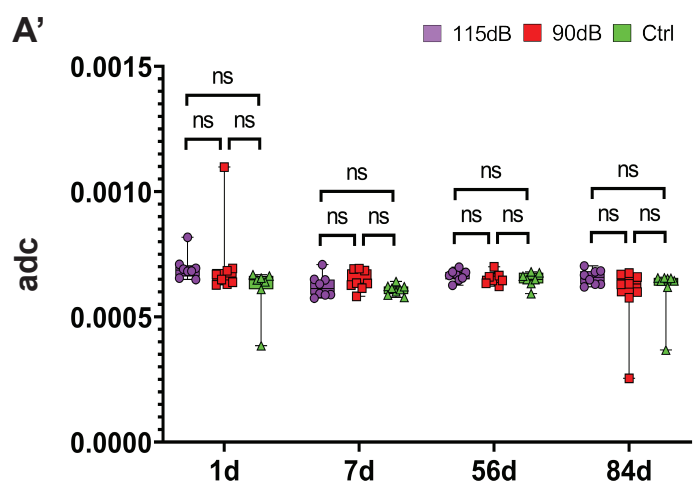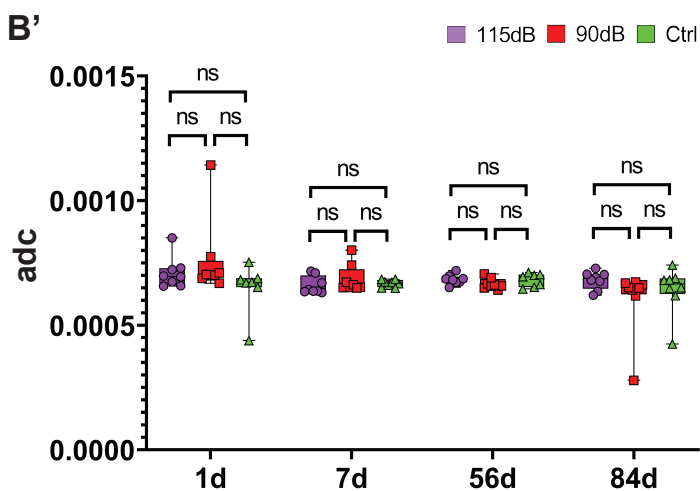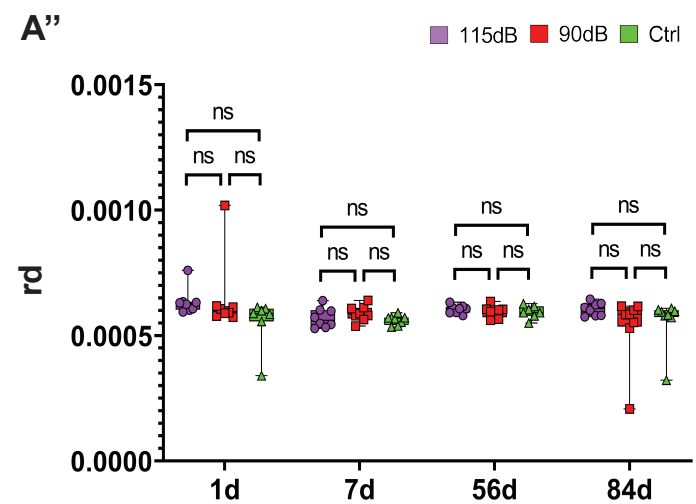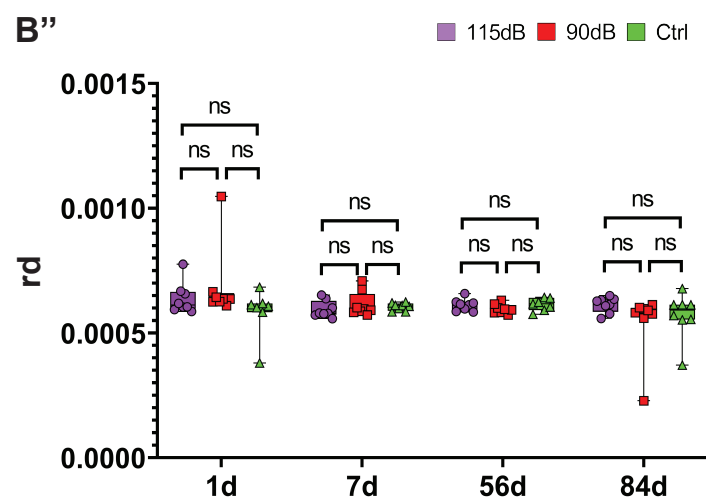

Supplement: Supplementary file 1 — Additional file1: Fig. S1. Microstructural connectivity changes after noise exposure. Diffusion MRI (dMRI) parameters were assessed in the central inferior colliculus (CIC; left panels, A–A″) and the ventral medial geniculate body (MGV; right panels, B–B″) at different time points following noise exposure. Group comparisons revealed no statistically significant changes in axial diffusivity (AD; A, B), apparent diffusion coefficient (ADC; A′, B′), or radial diffusivity (RD; A″, B″) in either region across conditions and time points (p > 0.05 for all comparisons). Data is presented as mean ± standard deviation (ns = not significant, ***: p < 0.05, **: p < 0.01, ***: p < 0.001). [file 40478_2026_2252_MOESM1_ESM.pdf]
